# Factors influencing digital review of pathology test results in an inpatient setting: a cross-sectional study

**DOI:** 10.1093/jamiaopen/ooaa003

**Published:** 2020-03-17

**Authors:** Robert Challen, Krasimira Tsaneva-Atanasova, Tom Edwards, Luke Gompels, Mark Dayer, Martin Pitt, Leon Danon

**Affiliations:** o1 EPSRC Centre for Predictive Modelling in Healthcare, University of Exeter, Exeter, Devon, UK; o2 Taunton and Somerset NHS Foundation Trust, Taunton, Somerset, UK; o3 The Alan Turing Institute, British Library, London, UK; o4 NIHR CLAHRC for the South West Peninsula, St Luke’s Campus, University of Exeter Medical School, Exeter, UK; o5 Data Science Institute, College of Engineering, Mathematics and Physical Sciences, University of Exeter, Exeter, UK

**Keywords:** test result follow-up, quality improvement, clinical workflow, data quality, laboratory informatics

## Abstract

**Background:**

Delay or failure to view test results in a hospital setting can lead to delayed diagnosis, risk of patient harm, and represents inefficiency. Factors influencing this were investigated to identify how timeliness and completeness of test review could be improved through an evidence-based redesign of the use of clinical test review software.

**Methods:**

A cross-section of all abnormal hematology and biochemistry results which were published on a digital test review platform over a 3-year period were investigated. The time it took for clinicians to view these results, and the results that were not viewed within 30 days, were analyzed relative to time of the week, the detailed type of test, and an indicator of patient record data quality.

**Results:**

The majority of results were viewed within 90 min, and 93.9% of these results viewed on the digital platform within 30 days. There was significant variation in results review throughout the week, shown to be due to an interplay between technical and clinical workflow factors. Routine results were less likely to be reviewed, as were those with patient record data quality issues.

**Conclusion:**

The evidence suggests that test result review would be improved by stream-lining access to the result platform, differentiating between urgent and routine results, improving handover of responsibility for result review, and improving search for temporary patient records. Altering the timing of phlebotomy rounds and a review of the appropriateness of routine test requests at the weekend may also improve result review rates.

## INTRODUCTION

Large numbers of tests are undertaken in hospitals, and many healthcare professionals are involved in the care of a patient from admission to discharge. There is evidence that some test results will never be reviewed, or if reviewed, never followed up on, in both primary and secondary care.^1–3^ Estimates of the frequency of the failure to follow-up on test results are very variable, depending on setting,^2^^,^^4^^,^^5^ and the measurement methodology, but thought to occur somewhere between 1% and 22.9% of inpatient admissions,^1^ and this is more frequent where there is a transfer of care between care settings. Other studies (summarized by Callen et al^2^) found even wider variation, with inappropriate or incomplete follow-up for 6.8% of abnormal test result alerts in 1 study, to 62% of abnormal glucose screening tests results in another study. This variability is in part due to differences in clinical context, but also the exact nature of the definition of follow-up in these studies. Missed test results are found to be particularly problematic if there are test results which are pending after a patient is discharged.^6–10^ The failure to review test results represents not only a certain degree of inefficiency, but also a potential clinical safety issue resulting in missed or delayed diagnoses,^1^ for example, being a contributory cause for delayed diagnosis and treatment of lung cancer, and hepatitis C, among others.^6^^,^^11^

Investigations into the introduction of a mandatory test result acknowledgement system in a maternity unit in Australia^12^ showed a wide variation in the length of time it took for clinicians to review and acknowledge test results. They found that the mean time it took for a newly available blood test result to be viewed was 19.1 h, but the median was just 3 h, whereas radiology results were reviewed on average 47.7 h after being published, with a median of 20.8 h,^12^ suggesting that the distribution of time to view is heavily skewed. This variability can also contribute to a delay in diagnosis, and therefore action, as clinicians must first become aware of the abnormal result. The effect of directly alerting clinicians to abnormal pathology test results has been investigated in different studies^9^^,^^13^^,^^14^ and, taken together, a reduction in the length of time taken for initiation of corrective therapy was seen, by reducing the delay before the clinician becomes aware of an abnormal result, or by making the clinician aware of abnormal results after the patient has gone home.^7^^,^^9^

This article describes a retrospective cross-sectional study of the clinical use of electronic pathology result review software in Taunton and Somerset NHS Foundation Trust (TSFT). This aims to describe the people, process and technology factors found to influence both the timeliness and the completeness of clinical review of test results. The study focused on a set of test results for which the overall completeness of review of test results is broadly in line with research above,^2^ but with a focus on how this is affected by different system and workflow factors. The purpose of this is to identify how timeliness and completeness of test review could be improved through an evidence-based redesign of the use of clinical test review software within clinical workflow. This is relevant to people who design or implement software for pathology test review in a hospital setting. This study also serves as a baseline for assessing future changes to the software for pathology result review in TSFT.

The purpose of this article was not to determine the clinical significance of delays or failure to view test results, as there are numerous factors that affect clinical outcome that are not controlled for in this study, not least being that viewing a test result does not necessarily imply that an action has been taken as a result.

## METHODS

### Setting

The study was conducted on clinical tests from inpatients in Musgrove Park Hospital, which is part of TSFT. It is a district general hospital, providing care to a population of over 340 000. It also provides some specialist services for the whole of Somerset, making the catchment population around 544 000. The hospital has over 700 beds, 30 wards, 15 operating theaters, a fully equipped diagnostic imaging department, and a purpose-built cancer treatment center.^15^ TSFT shares its pathology services with all the hospitals and primary care providers in Somerset.

### Information governance

The data required for this audit are routinely automatically collected as part of the pathology viewer software audit log. The data were fully de-identified at source before being analyzed by TSFT staff on computers within the hospital’s secure data center. As an internal audit of TSFT operations using nonidentifiable data it did not require patient consent. It was reviewed and approved by the hospital research and development office and Caldicott guardian (information governance lead). No patient identifiable information was retained by the data extraction.

### Dataset

When a result is directly viewed on the system, the pathology viewer software records the time that the result was accessed in an audit log. There is also an indirect method for viewing test results in a timeline-based “grid view,” in which all of the most recent results are presented simultaneously. This indirect method of viewing results is also logged by the system, but not at the individual test result level. Based on feedback from clinical users of the system, the assumption was made that clinical review of results using the grid view is equivalent to the direct review of all test results released in the preceding 24 h.

The source database integrates results from many laboratories, and radiology departments, stemming from requests from many primary and secondary care providers. As such the data quality of patient records has issues, particularly in terms of the creation of temporary duplicate patient records which require subsequent merging. Such records may be created if a patient’s contact details have changed or are miskeyed, or if a patient is admitted in an emergency where incomplete demographic details are available. During this subsequent merging process test results are reassigned to the canonical patient identifier when possible. Prior to the merge, test results filed under temporary patient record may be less easy to find than those correctly filed from the outset. Record merging may happen before, during, or after results become available on the system, but the results are only reassigned after they have been published. The reassignment of a test during this merge process was extracted as an experimental variable and is an indicator of the initial data quality of the patient record.

### Selection criteria and study size

The existing laboratory results viewer system contains results from the last 10 years for the whole of Somerset, involving inpatients and outpatients over multiple hospital sites and primary care providers. These were limited to tests that had been requested from inpatient wards, and emergency ambulatory care in TSFT between September 1, 2014, and September 30, 2017, during which no major changes occurred in either the test requesting and review processes, or in the laboratory test result review platform itself. The set was constrained to the 1 770 775 million biochemistry and hematology reports that were reported as abnormal on the system, and for these results, there were no missing data items. Abnormal results are visible as such on the test review system and such abnormal results represent investigations that should have clinician review. Finally, unlike radiology reports which are available on the picture archive and communications platform, biochemistry and hematology test results are only available digitally through the results review platform. They are less widely reviewed via paper reports in TSFT than microbiology, as the paper reports are only distributed some hours after the digital report is available. This delay makes the paper hematology and biochemistry reports less clinically useful than digital reports, but not so for microbiology given the longer turn-around times associated with cultures, and comparatively more telephone alerting of clinicians to abnormal results.

### Data analysis

**Figure 1.**
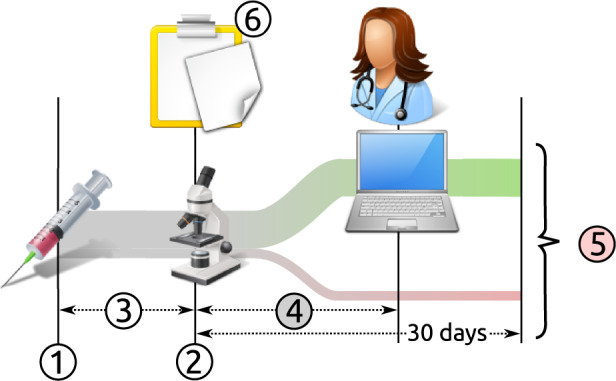
At time point (1) a specimen and associated request starts being processed at the laboratory. At (2) the result is made available on the electronic results system. The processing time (3) depends on various factors including the type of test. After a period of time (4) a result may be viewed by one or more clinicians—the “time to view.” Some results are not reviewed digitally or reviewed very late (5) (30-day cut-off was applied)—these results are deemed to be “unviewed.” Test results, however, may have been viewed through parallel routes such as paper reports (eg, microbiology) (6) or second dedicated systems (eg, radiology).

Dependent and experimental variables were extracted from the source database using a custom structured query language routine. The main dependent variables investigated were the time for the first clinician to view a test result after it becomes available on the system (time to view) and the proportion of tests which were not digitally viewed on the pathology results viewing system within 30 days (unviewed tests) which included 99.9% of all test views. These outcomes are described in Figure 1. Qualitatively the timeliness of test review is related to the overall distribution of the time to view, but is essentially quantified here as the median of the time to view all the tests in a given sample (Timeliness = median of time to view a test result). Completeness of test result review for a given sample can be quantified as the complement of the unviewed tests (Completeness = 1 − Number of tests results not viewed/Total number of test results). Test results were described at the level of the clinically ordered test battery, so this study regards the combined test of “Full Blood Count” as a single test, rather than the individual component “Hemoglobin level.” Only the most up to date revision of a test result was included.

The study stratified the sample by a number of different experimental variables and visualized the differences in time to view and percentage of test unviewed after 30 days by these experimental variables. Among many other factors considered as experimental variables, we report here on temporal variation, the detailed test type of the result, and the test reassignment status as defined above. Temporal variation was investigated using the time within the week when the test result was published as an experimental variable and it was hypothesized that tests may be reviewed less quickly when staffing levels are lower overnight, or during the weekend. The detailed type of test result was selected as clinical tests have different clinical impacts and it was expected that some test types will be monitored more closely by clinicians. Test reassignment was investigated as a marker of data quality of the patient record. It is expected that reassignment of tests to different patient identifiers are a proxy measure for the ease with which clinicians find test results, and hence would be related to how many results are missed.

All data were analyzed and visualized using R.^16^ Correlation strengths are estimated with Cramér’s V coefficient^16^ and Pairwise Wilcoxon tests.^17^ Distributions of time to view were compared with Kruskal–Wallis tests.^18^ Instantaneous publish and view rates were calculated using piecewise polynomial fitting to cumulative rates using a Savitzky–Golay filter.^18^ Continuous distributions of median time to view were calculated from a rolling 2 h window, and error estimates median time to view estimated using case resampling bootstrapping.^17^ Estimates of error in unviewed tests were determined by the size of the group, assuming a binomial distribution.

## RESULTS

### Sample characteristics

**Figure 2.**
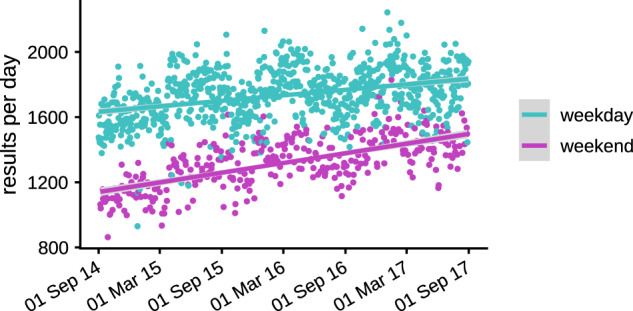
The rate of abnormal biochemistry and hematology test results per day during the study period.

There were 1 770 775 abnormal biochemistry and hematology test results released during the study period (approximately 1600 per day). Figure 2 shows the difference in daily test result rates between weekdays and weekends and demonstrates a steady increase over the study period.

**Figure 3.**
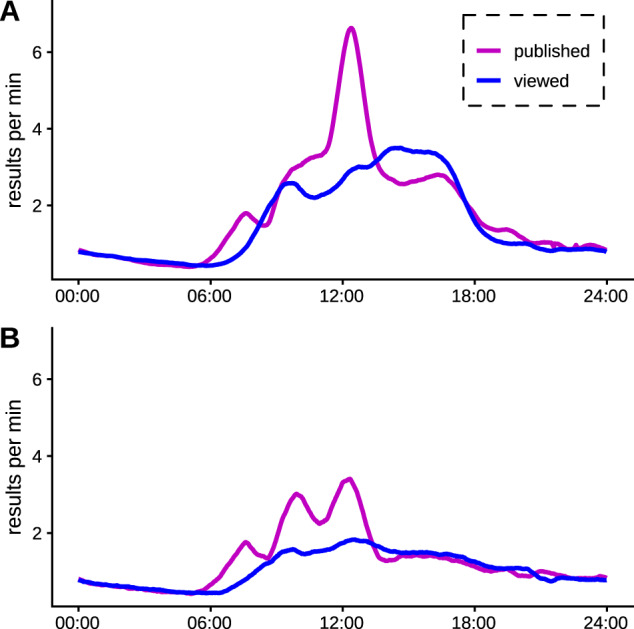
Publish and view rates over the course of the day for test results, for weekdays (A) and weekends (B).

Figure 3 shows the rates of test publication and test review during the day, split between weekday and weekend. A peak of activity in test publication is noted at midday. The weekend test publication pattern could represent a similar pattern with a smaller midday peak.

Of the test results that were published, 7.69% (*N* = 136 175) were subsequently reassigned to another patient record during a duplicate record merge process, as described in the “Methods.” This leaves 92.31% (*N* = 1 634 600) which were correctly assigned to the canonical patient record at publication.

### Summary of dependent measures

**Figure 4.**
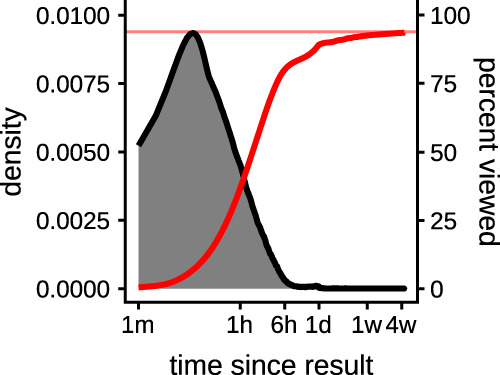
The distribution of the time to view a result within the first 12 h of it becoming available (grey) shows a heavily skewed distribution with a long tail. The cumulative proportion of tests viewed is shown in red. Time is plotted on a logarithmic scale.

The time to view abnormal biochemistry and hematology test results is shown in Figure 4. The distribution is heavily skewed with a median of 89.8 min (interquartile range 33.9–213.8 min). This median has stayed constant during the study period (see Supplementary Table S1). Over the 30-day period after each of the 1 770 775 abnormal biochemistry and hematology test results were made available, 1 662 067 results were viewed on the digital platform (93.9%) with the remaining 108 708 (6.1%) of all test results being unviewed at 30 days (Figure 4). An unknown proportion of these 108 708 test results will have had clinical review of the paper copy. In the logarithmic time plot in Figure 4, 3 behavioral regimes are observed (1) a fast, exponential rise to a peak value at around 30 min, (2) an exponential decline between 30 min, and (3) a long tail with a small second peak of test viewing activity at 24 h.

### Detailed test result type

**Figure 5.**
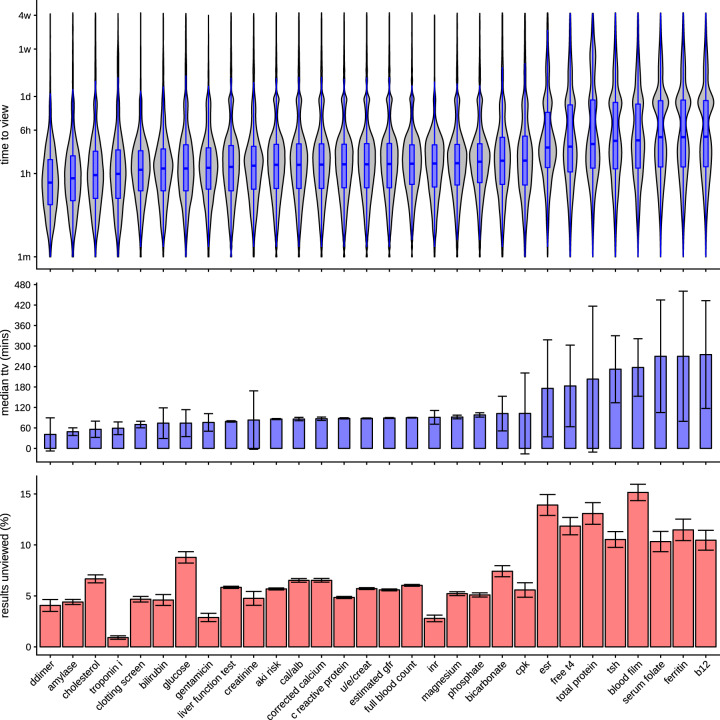
Time to view distribution (top), median time to view (middle), and proportion unviewed (bottom) for the 30 most common tests. The top violin plot shows the distribution of time to view in the first 30 days from publication on a logarithmic timescale, for each test. In the middle plot, the same median is shown with 95% confidence intervals for the median calculated using a bootstrap estimation. In the lower plot, the absolute proportion of tests that are unviewed is shown with 95% confidence intervals determined by the size of the group, assuming a binomial distribution.

The 30 most frequently performed tests are shown in Figure 5 and a qualitative difference is apparent between tests such as D-dimer, or troponin I assays, which have a high clinical impact in the short term, and tests such as ferritin, or vitamin B12 levels, abnormalities of which have longer-term sequelae, and management. Both the speed and the likelihood that a clinician is to review a result varies depending on the test type. Correlation strengths are estimated with Cramér’s V coefficient^19^ for the relationship between test type and whether a result has been viewed is 0.585. A Kruskal–Wallis^20^ test demonstrated a significant difference between distributions of time to view (*χ*^2^ = 20 476, *P* < 2.2 × 10^−16^). Pairwise Wilcoxon tests^21^ demonstrated some correlated test types, where the results of a test are frequently reported together (eg, Cal/alb ratio and corrected calcium).

### Time of the week of test result publication

The rate at which tests are published and reviewed (Figure 4) demonstrated a daily rhythm of the test request and review process.

**Figure 6.**
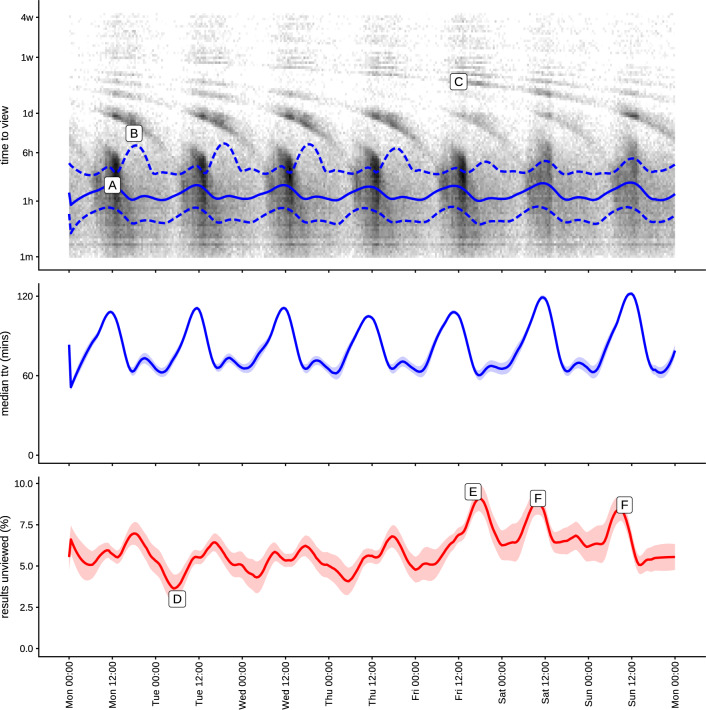
Time to view distribution (top), median time to view (middle), and proportion unviewed (bottom) for the tests with relation to the time of the week. Darker colors of the top plot represent higher number of tests released at a given time of the week and how those are distributed over the time taken to view them. The blue lines showing the median (solid) and interquartile ranges (dashed) of time to view on a logarithmic timescale. The banding pattern represents results being reviewed on subsequent days. In the middle panel the same median time to view is shown in a linear time plot with very narrow 95% confidence intervals estimated using bootstrapping. The bottom plot shows the percent of tests that are not viewed, broken down by when they are published. We see high rates of test review at point (A), for test results published at midday on Monday. Results published later in the day may not be seen until the following day (B). This effect is more pronounced on Friday where results may not be seen until the following Monday (C). Tests are most likely to be viewed if they are published early on Tuesday (D) and least likely to be viewed if they are published on Friday evening (E) or during the day in the weekend (F).

The impact of this daily pattern on the time taken to view tests and the likelihood a test is not viewed was investigated and the results are shown in Figure 6. The density plot in the top panel has notable features. From Monday to Friday, the bulk of test results are viewed during the first six hours after becoming available, but the delay in reviewing a test increased throughout the morning. A test released at midday took twice as long to be reviewed as those made available at 6 Am (A). Through the afternoon and overnight, the delay reviewing a result dropped again, but the chance of the test being reviewed the following day increased (widening the interquartile range of time to view) (B). On Friday, the pattern is slightly different in that the results that became available in the afternoon and not seen the same day were more likely to be viewed on Monday morning (C).

**Figure 7.**
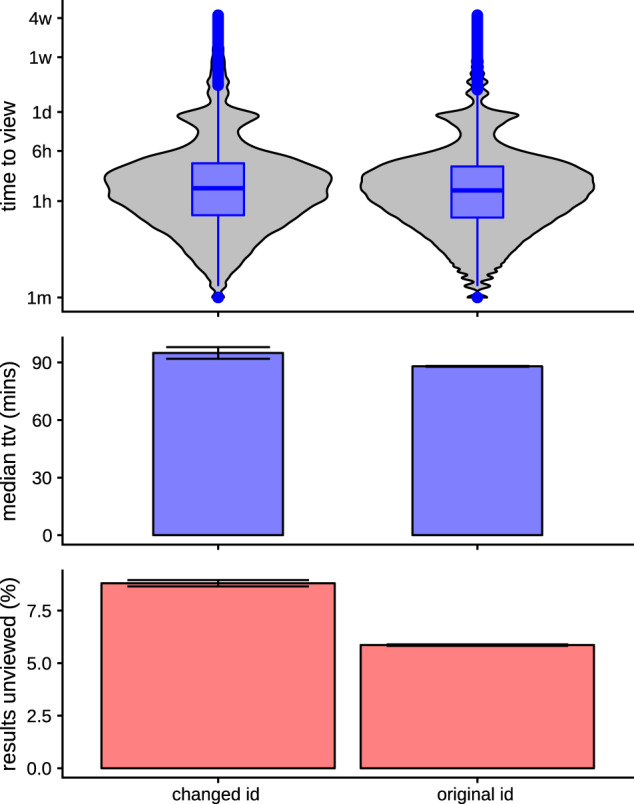
Time to view distribution (top), median time to view (middle), and proportion unviewed (bottom) for the tests that have had their patient identifier changed versus those that retain their original patient identifier. Tests that have been updated and rematched to a “better” patient record are less likely to be viewed. 95% confidence intervals are shown on the graph. *P*-values for both comparisons are smaller than numerical precision of the calculating software due to the large numbers of tests involved.

The variation of the median time to view over the week is shown in the middle panel of Figure 7 for completeness as the linear plot better demonstrates the effect size and the relative errors of our estimates. It shows the increasing delay in test result viewing over the course of the morning, recovering by mid-afternoon, followed by a smaller second increase at the end of the afternoon as a proportion of results were left until the next day.

The proportion of tests that are not viewed is shown in the bottom plot of Figure 6. Tests conducted overnight when staffing levels were low were more likely to be looked at than those in the middle of the day (point D). As with the time to view, the chance of a test being missed increased if it was released later in the afternoon during the week. The most significant increase in unviewed tests occurred on Friday afternoon when a released result has just under 10% chance of not being seen (E). On the weekend the pattern was slightly different, with tests that were performed in the middle of the day being less likely to be viewed (F).

### Test result reassignment

As described in the “Methods” above, test result reassignment may be an indication that a test result was difficult to find and less likely to be reviewed. The patient identifier was revised in 136 177 out of 1 770 775 of test results (7.69%) of which only 2386 (1.78%) represented revision to a completely different patient, for example, due to error ordering or processing the test. The remainder was revisions to a different identifier of the same patient and represents a data quality issue encountered during integration of new test results. Although this had only a small effect on the time to view the results, there was a significant effect on the proportion of results not viewed, with an additional 4% of the revised tests not being looked at within 30 days, compared to those which are not revised (see Figure 7).

## DISCUSSION

### Principal findings

For inpatients within TSFT, blood tests are typically performed during admission and throughout the patient’s stay as determined by the clinical team. Dedicated phlebotomists generally pick up requests, and draw blood for blood tests during the early part of the morning on both weekdays and a limited service at weekends. These will usually not be urgent test requests, and the request for the test may have been made the night before. If an urgent test is required blood will be drawn by a member of the clinical team. This system explains the morning peak in the volumes of tests pattern shown in Figure 3 when a high proportion of test results will be from routine requests.

Typically, particularly for general inpatients, the clinical team will conduct a ward round during the morning during which they will review patients, including the available blood test results if possible. This ward round is largely conducted using paper notes, and during this time the clinical team has variable access to desktop PC bound resources, including the laboratory test review software. Ward rounds generally finish during the course of the morning, after which the junior members of the clinical team have better access to digital systems.

Test results may be reviewed by doctors, midwives, or any other clinical staff caring for the patient. However, the bulk of the results review, particularly of routinely requested tests from that day, will be performed by the junior clinical staff, typically during the afternoon. We believe that some of the increased delay in test result review in the middle of the day seen in Figures 3 and 6 is a result of the limited access that junior members of the clinical team have to the results platform during the ward round. Seriously abnormal results may be phoned through to ward staff, who will then alert a clinician to the result, who in turn will typically review the patient’s results.

Outside of weekday working hours, the hospital runs a shift system for clinical staff.^22^ This will typically involve a handover of clinical care around 5 Pm to an evening shift, and again at approximately 10 Pm to an overnight shift. In general, these teams will only be involved with acutely sick patients and will not consider routine test results. This handover of responsibility, and shift away from routine, to urgent care, is likely to be the main driver for the increased delay for test results that are published later in the day seen in Figure 6.

The possibility of a strong effect of routine versus urgent testing on both completeness and timeliness of test result review is supported by the analysis of detailed test type shown in Figure 5. Tests for significant acute medical conditions such as D-dimer tests for thrombosis, or troponin tests for myocardial infarction, are more rapidly and completely reviewed than tests that are long term in nature, such as investigations for iron deficiency anemia. One possible conclusion is that investigation of longer-term health problems in inpatients is less efficient than the investigation of an acute health problem, but this also points to some prioritization of clinical review of test results that are urgent in nature.

Finally, this study found evidence in Figure 7 that data quality has a significant effect on the result review, in that test results which have had to be reassigned to a duplicate patient identifier during a merge, were less likely to be reviewed. This is evidence that efforts should be directed at not only improving data quality, but also that further work is needed to understand what mitigations need to be in place for finding hard to identify and temporary patient records.

### Limitations

This study has several drawbacks due to the nature of information available and quality of some fields in the source database. Particularly information about the identity of test requesters reviewers was not well standardized, and neither was information about the precise location of ordering and reviewing results.

As a retrospective observational data study we were unable to collect hard data about the workflow of test requesting, sample collection, test reviewing, and clinical ward rounds. Instead all the assumptions about the nature of the workflow have been reviewed for accuracy by 6 independent TSFT staff, consisting of both frontline clinicians and members of the digital implementation team in TSFT involved in the deployment and maintenance of the digital test result platform.

Evidence suggests that there is a strong effect of the patient’s admission and discharge on the timeliness and completeness of test result review.^6–10^ A linked patient admission, discharge, and transfer dataset was not available and so this study is unable to confirm previous findings about the significance of tests that are pending at discharge.^5^^,^^7–9^ As patient discharge typically occurs in the later part of the day, a strong relationship to patient discharge could indirectly explain some of the observed patterns. This would warrant investigation as one possible confounder for the temporal patterns observed. Ineffective follow-up of test results pending at discharge has previously been shown to be a cause of diagnostic delay,^2^^,^^6^ but as this was not available for analysis it should be a focus of further work.

As indicated in Figure 1, the digital review of test results is not the only mechanism through which results are viewed. Biochemistry and hematology results are printed on paper, delivered to requesting location, where the paper results may be reviewed, but these are subsequently shredded and not filed in the patient record. Paper-based review is quite variable across the hospital and is not recorded, but it should be expected that the estimate of unviewed tests presented here was at the upper end of the true value. Similarly, a test result can be clicked but not actually reviewed due to some distraction in practice, or a test result may need to be viewed multiple times to complete the review process.

Because of issues associated with parallel review processes we did not consider microbiology and radiology test results in our analysis. The complexity of result review in microbiology is described by Bruins et al^23^ and this analysis focused on the simpler problem of biochemistry and hematology test results. However, many of the examples in the literature that describe the clinical impact of missed test results focus on microbiology and radiology^5^^,^^9^ and hence this reduces the comparability of our findings to previous studies.

Viewing a test result does not imply that appropriate clinical action has been taken, or that the viewing clinician has taken responsibility for follow-up. There were no clinical outcomes outside of lab results available in our dataset; investigation was conducted to see whether repeat testing could be used as a marker for clinical action as in Lin and Moore,^24^ but this was uninformative. It has not been possible to follow-up on the clinical significance of abnormal test results, and there is no simple way to determine whether an unviewed test could have affected the outcome, and whether an unviewed test result itself would have provided useful information to a clinical decision-making process.

### Suggestions for improvement

It is to be expected that many of these findings, particularly around workflow, are specific either to TSFT itself or to hospitals in the National Health Service (UK). However, the findings suggest some improvement can be made in the efficiency of test review:

1. The competing demands for a clinician’s attention during the morning ward round and potential difficulty accessing a static desktop PC bound resource while at the bedside could be improved by providing access to test results as soon as they become available through mobile devices.
2. Test ordering and review systems should consider differentiating between urgent and routine requests in both alerting the clinician of the availability of a result and informing them of unexpected abnormality.
3. Further technical measures could be adopted to better alert clinicians to abnormal results at times of the day when test are likely to be missed, for example, by more proactive alerting clinicians to abnormal results on Friday afternoons, or by requiring mandatory sign off of abnormal results.^12^
4. Further improvement might be expected if the clinical review software was enhanced to allow better handover of clinical responsibility for review of abnormal test results between clinicians working different shifts. Suboptimal transfer of care between shift working teams within the hospital has previously been identified as an area of high risk.^25–27^
5. Improvements in patient record search prior to test ordering may improve data quality. This may reduce the number of tests reported under suboptimal duplicate patient records and which are subsequently lost in the system. However, better search for temporary records in the result viewing platform is also warranted.
6. Test result review would be more efficient if fewer results were pending during the clinical ward rounds. This might be achieved, for example, by changing the timing of the phlebotomy ward rounds to ensure results are available before clinical ward rounds.
7. Spikes in the rates of unviewed tests observed at the weekend could represent abnormalities in routine monitoring tests requested by the regular team, which are not being picked up by the weekend team, whose focus is on urgent care. These tests are arguably of low clinical value. A focus on appropriate test requesting at the weekends may be indicated.

## CONCLUSIONS

This study presents a detailed analysis of a large sample of electronic test results. The volume enables a closer look at workflow patterns during the day than has been presented previously, and this shows several patterns which are thought to be related to whole system workflow, information availability and handover of clinical responsibility, which have not, to our knowledge, been demonstrated before in the literature. To some degree the effect of shift handover may be explained by a transition from urgent to routine care, as previously described.^28^

The study identifies that the data quality of the patient record can influence the ease of locating a test result and increase the likelihood that a result will go unreviewed. This is a novel finding that is, so far, specific to TSFT but which would be useful to validate in another setting. Taken together these findings allow the recommendation of various changes to the systems and workflow of test review in TSFT as outlined above and the authors believe these are of relevance to other hospitals.

TSFT is in the process of implementing a mobile device-based pathology results viewer. The effect of this intervention on the workflow factors presented here may be significant as test results will be more easily available, and this will be monitored using this study as a baseline. We anticipate the recommendations presented in this study will inform future technical development and implementation of test result review platforms.

This is a retrospective observational study of a single site and our conclusions are limited by this. It would be useful to repeat this study in different hospitals to compare the results. It would be particularly valuable to look at a dataset that included the patient’s admission, discharge, and transfer details to investigate the effect of handover and tests pending at discharge better. This study is designed to be a baseline to provide evidence for quality improvement initiatives and technical enhancements to the laboratory test reporting platform. Further studies should focus on periods before and after such interventions to more rigorously test the improvements suggested above.

## FUNDING

RC and KT-A gratefully acknowledges the financial support of the EPSRC via grant EP/N014391/1 and NHS England, Global Digital Exemplar Programme. LD gratefully acknowledges the financial support of The Alan Turing Institute under the EPSRC grant EP/N510129/1.

## AUTHOR CONTRIBUTIONS

All authors discussed the concept of the article and RC wrote the initial draft. KT-A, TE, LG, MD, MP, and LD commented and made revisions. All authors read and approved the final manuscript. RC is the guarantor. The views presented here are those of the authors and should not be attributed to TSFT or the GDE.

## ETHICS APPROVAL and CONSENT TO PARTICIPATE

As an internal audit of TSFT operations using nonidentifiable data, this study did not require patient consent and has been exempted from formal NHS Health Research Authority approval and review by a research ethics committee. It was reviewed and approved by TSFT research and development office and Caldicott guardian (information governance lead).

## AVAILABILITY OF DATA and MATERIAL

The datasets generated and/or analyzed during the current study are not publicly available due to the remote possibility of targeted patient re-identification through a linkage attack. Limited data may be available from the corresponding author on reasonable request, and review by the information governance and ethics teams.

## SUPPLEMENTARY MATERIAL

Supplementary material is available at *Journal of the American Medical Informatics Association* online.

## Supplementary Material

ooaa003_Supplementary_Data
