# Supplementary material for: Factors influencing digital review of pathology test results in an inpatient setting: a cross-sectional study
Source: JAMIA Open. 2020 Mar 17;3(2):290–8. doi: 10.1093/jamiaopen/ooaa003 (PMC7382616; doi:10.1093/jamiaopen/ooaa003)
Supplement: ooaa003_Supplementary_Data [file ooaa003_supplementary_data.pdf]

SUPPLEMENTARY MATERIAL FOR FACTORS INFLUENCING DIGITAL REVIEW OF PATHOLOGY TEST RESULTS IN AN INPATIENT SETTING: A CROSS-SECTIONAL STUDY

Table S1 - the baseline coefficients for a linear models of the form $y = At + B$ for the daily estimates of time to view and proportion unviewed over the study period. σ is the residual standard error. The models are centred around the midpoint of the study period (and as such the intercept, B, is the mean for the study period). All measures are very slightly decreasing over time.

**variable**	**A**	**B**	**Adj R^2**	**σ**
Median time to view	-0.017 mins day^{-1}	89.8 mins	0.1216	14.21 mins
Lower quartile	-0.005 mins day^{-1}	33.9 mins	0.0658	6.157 mins
Upper quartile	-0.053 mins day^{-1}	213.8 mins	0.1676	37.59 mins
% tests unviewed	$-5.1x10^{-5}$ % day^{-1}	6.129 %	< 0.001	1.887 %
